# Exploring differential exon usage via short- and long-read RNA sequencing strategies

**DOI:** 10.1098/rsob.220206

**Published:** 2022-09-28

**Authors:** Dena Leshkowitz, Merav Kedmi, Yael Fried, David Pilzer, Hadas Keren-Shaul, Elena Ainbinder, Bareket Dassa

**Affiliations:** Life Sciences Core Facilities, Weizmann Institute of Science, Rehovot 76100, Israel

**Keywords:** alternative splicing, differential exon usage, long-reads, short-reads, embryonic stem cell, RNA-Seq

## Abstract

Alternative splicing produces various mRNAs, and thereby various protein products, from one gene, impacting a wide range of cellular activities. However, accurate reconstruction and quantification of full-length transcripts using short-reads is limited, due to their length. Long-reads sequencing technologies may provide a solution by sequencing full-length transcripts. We explored the use of both Illumina short-reads and two long Oxford Nanopore Technology (cDNA and Direct RNA) RNA-Seq reads for detecting global differential splicing during mouse embryonic stem cell differentiation, applying several bioinformatics strategies: gene-based, isoform-based and exon-based. We detected the strongest similarity among the sequencing platforms at the gene level compared to exon-based and isoform-based. Furthermore, the exon-based strategy discovered many differential exon usage (DEU) events, mostly in a platform-dependent manner and in non-differentially expressed genes. Thus, the platforms complemented each other in the ability to detect DEUs (i.e. long-reads exhibited an advantage in detecting DEUs at the UTRs, and short-reads detected more DEUs). Exons within 20 genes, detected in one or more platforms, were here validated by PCR, including key differentiation genes, such as Mdb3 and Aplp1. We provide an important analysis resource for discovering transcriptome changes during stem cell differentiation and insights for analysing such data.

## Graphical abstract

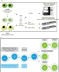

## 1. Introduction

High-throughput short-read sequencing transcriptional profiling (RNA-Seq) was pioneered in 2008, enabling quantitative transcriptome-wide surveys of gene expression and alternative splicing [1–4]. RNA-Seq has greatly expanded our knowledge of the transcriptome, providing reliable quantification and detection of differential expression at the gene level [5,6]. However, transcript level or isoform-based analysis is error-prone, since short reads cannot unambiguously resolve the connectivity between distant exons, particularly when alternative splicing generates multiple, partially redundant isoforms [6–9]. Isoform-based analysis requires a complete and accurate isoform construction and quantification of full-length transcripts as the basis for a confident differential splicing (DS) analysis. With the emergence of third-generation sequencing, it is now possible to sequence full-length transcripts in ‘one go’ and directly identify isoform structures thereby overcoming the challenges posed by computational assembly of short reads [10–13]. Oxford Nanopore Technology (ONT) directly sequences a native single-stranded DNA molecule, by measuring characteristic current changes as the bases are threaded through the nanopore by a molecular motor protein [14]. Using ONT technology, both cDNA and Direct RNA long reads can be sequenced [10–12,15–19]. In the Direct RNA approach, individual poly-adenylated RNA transcripts are directly sequenced, without recoding and amplification biases inherent in other sequencing methodologies. Yet, the relatively high long reads error rates, of above 10% for both direct RNA and cDNA sequences, complicate the detection of the transcript's exact exon structure [10,11,20,21]. Several computational and sequencing methods have been developed to overcome this challenge [22–24], yet all these methods are applicable only to cDNA.

Many studies have compared the transcriptome landscape between these long- and short-read sequencing technologies [10,11,13,15,16,25–27]. A study by Mehmood *et al.* using short reads has shown that exon-based methods generally performed better than the isoform-based methods [9]. Furthermore, studies have used long reads using the exon-based approach [27–30]. To characterize the strengths and remaining challenges in using long-read approaches, a community effort called the Long-read RNA-Seq Genome Annotation Assessment Project Consortium has been launched [31].

Here, we characterized the strengths and potential of both short- and ONT long-read sequencing platforms to explore transcriptomic changes during *in vitro* differentiation of mouse ESCs induced by retinoic acid (RA) [32,33]. Differentiation of embryonic stem cells (ESCs) is among the most dynamic processes in biology. Mouse ESCs, derived from the inner cell mass of mouse blastocysts, are pluripotent cells that have the capacity to differentiate into cell types of all three primary germ layers [34]. Regulation of ESC development, pluripotency and reprogramming is mediated by transcription factors [35], and involves transcriptome changes and isoform switching via alternative splicing [36–41]. Recently, long reads were used to study alternative splicing events during early embryogenesis [10,42].

In this study, RNA was collected before and after RA-induced ESCs differentiation, and sequenced via Illumina to generate short reads (RNA-Seq). In parallel, long-read sequencing was performed using ONT technology, generating both cDNA and Direct RNA long reads. Our bioinformatics analysis aimed to explore changes using three strategies: the gene, isoform-based and exon-based levels. We demonstrated that detection of differential exon usage (DEU) events, developed for short reads, was also applicable with long reads, and that the three sequencing platforms predictions complement one another and are reliable as validated by PCR. In addition, we provide an important sequence and analysis resource for discovering transcriptome changes occurring during stem cell differentiation.

## 2. Results

### 2.1. Study design and data processing

**Figure 1.**
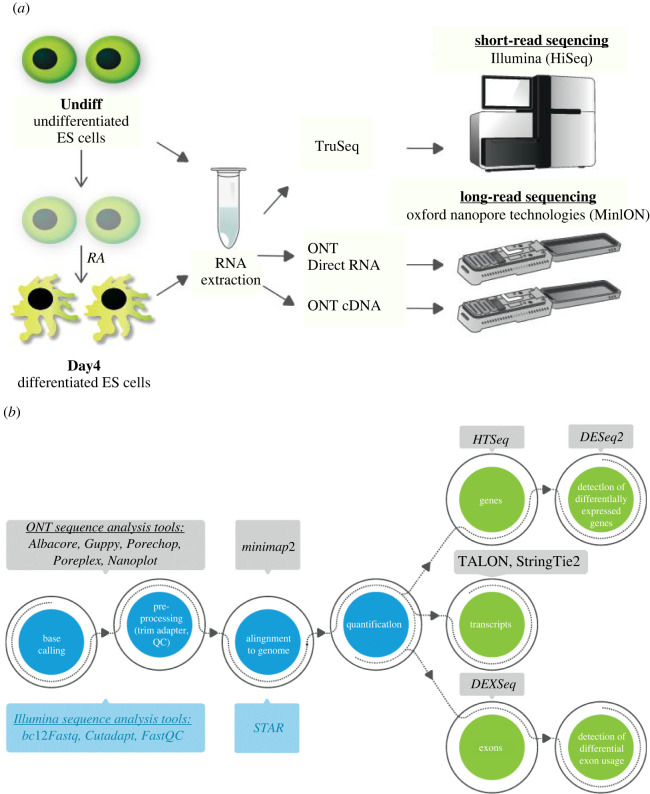
(*a*) A schematic workflow of the experimental design. Mouse embryonic stem cells were grown for 4 days in mES growth medium (Undiff), and then for an additional 8 days, of them 4 days with retinoic acid (RA) (Day4). RNA was extracted and sequencing libraries were prepared for short-read sequencing with Illumina (TruSeq library), and long-read sequencing with Oxford Nanopore Technologies, either cDNA or Direct RNA library platforms. (*b*) The bioinformatics analysis pipeline. Raw sequences were pre-processed and aligned to the mouse genome using tools for either short or long reads. Expression was quantified at gene, transcript and exon levels, to identify differentially expressed genes (DEGs) and differential exon usages (DEUs) in Undiff versus Day4 samples, using the same tools for all platforms.

This study focused on detecting transcriptome changes during ESC differentiation. Towards this aim, total mouse RNA was extracted from embryoid body duplicate samples before (Undiff) and after (Day4) differentiation with RA (figure 1*a*; see also Material and methods). RA plays multiple roles in the nervous system, including induction of neural differentiation, axon outgrowth and neural patterning [32]. The RNA was used for short-read sequencing with the TruSeq library and Illumina platform, and for ONT long-read sequencing technology with both cDNA and Direct RNA kits (named herein as platforms: Illumina TruSeq, ONT cDNA and ONT Direct RNA, respectively). The yield of short reads was around 50 M (paired-end fragment sequenced) per sample. The ONT MinION flow cell yield was around 3.5 M for cDNA, and around 1 M for the Direct RNA (table 1). ONT and Illumina dataset sequence processing required different bioinformatics tools, as demonstrated in figure 1*b* and described in the Material and methods section. Analyses starting at the stage of the mapped reads from all three platforms (Illumina TruSeq, ONT cDNA and ONT Direct RNA) were conducted using the same procedures, to compare sequence quality features, and detected expression at the gene, transcript and exon levels, as well as the ability to detect differentially expressed genes (DEGs) and DEUs.

**Table 1.** Statistics on sequencing processing steps.

technology	differentiation state	replicate	total number of reads/fragments in millions	N50 of read length (no. bases)	% alignment error rate	% primary/uniquely aligned reads/fragments^a^	read bases aligned in millions	total no. of reads/fragments mapped uniquely to genes	total no. of genes expressed^b^	total no. of TALON transcripts expressed^c^	total no. of StringTie transcripts expressed^d^	total no. of exons expressede
ONT Direct	Undiff	replicate1	0.63	1568	14.30	98.91	675.55	348 457	10 399	16 937	13 462	152 231
ONT Direct	Undiff	replicate2	1.64	1496	14.60	96.58	1705.26	907 489	11 822	19 399	15 956	186 248
ONT Direct	Day4	replicate1	0.46	1783	14.30	100.00	582.69	283 461	10 631	16 830	13 728	158 808
ONT Direct	Day4	replicate2	1.31	1612	14.10	96.97	1447.48	737 569	12 322	19 775	16 908	194 115
ONT cDNA	Undiff	replicate1	1.96	944	12.50	92.74	1365.52	948 657	10 793	19 425	12 954	146 250
ONT cDNA	Undiff	replicate2	5.53	961	11.40	97.19	3085.33	2 088 592	12 240	21 347	15 821	105 947
ONT cDNA	Day4	replicate1	2.82	1096	12.40	85.01	2090.33	1 318 178	11 884	17 995	13 840	163 912
ONT cDNA	Day4	replicate2	3.41	1238	10.00	99.97	3278.57	2 077 327	13 180	20 443	16 576	130 377
Illumina TruSeq (PE 101)	Undiff	replicate1	49.06	202 *fragment	0.28	86.86	8513.55	36 674 893	17 359	34 493	24 557	262 506
Illumina TruSeq (PE 101)	Undiff	replicate2	44.18	202 *fragment	0.28	85.57	7090.89	32 099 074	17 219	34 199	24 212	256 229
Illumina TruSeq (PE 101)	Day4	replicate1	43.53	202 *fragment	0.28	88.64	7717.00	34 956 489	16 828	32 951	23 374	259 039
Illumina TruSeq (PE 101)	Day4	replicate2	59.65	202 *fragment	0.28	88.53	8522.77	47 730 944	17 170	31 968	23 381	266 617

^a^Primary aligned reads for ONT platform, and uniquely aligned reads for Illumina.

^b^Total no. of genes expressing more than one read, quantified by HTSeq (out of 24 421 genes in GENCODE).

^c^Total no. of TALON assembled transcripts quantified by StringTie2 with FPKM greater than 1 (out of 97 903 transcripts).

^d^Total no. of StringTie2 assembled transcripts with FPKM greater than 1 (out of 147 769 transcripts).

^e^Total no. of exons expressed by more than 1 read (out of 437 918 exons).

### 2.2. Inter-platform comparison of aligned read characteristics

**Figure 2.**
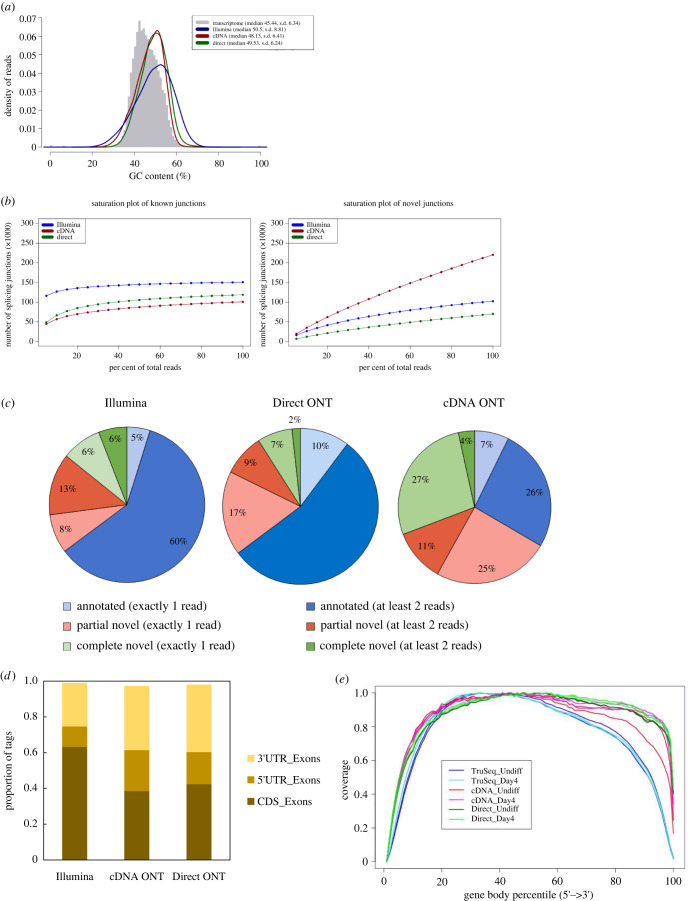
Comparison of aligned read quality features between Illumina TruSeq, ONT cDNA and ONT Direct RNA platforms. Panels (*a–d*) relate to averaged values of Day4 replicates. (*a*) GC content distribution of reads across the platforms. In grey is the theoretical distribution calculated for all transcripts (Transcriptome). Median and standard deviation (s.d.) are noted in the legend. (*b*) Saturation of known and novel splice junctions. The plot reveals saturation by resampling 5%, 10%, 15% etc. of the total alignments. (*c*) The proportions of reads, detected in one of the six categories of junction annotations, supported by a single read, or by at least two reads, and defined as Annotated (known) (both 5′ and 3′ splice sites are annotated by reference gene models), Partial novel (one of the splice sites 5′ or 3′ is novel) or Complete novel (both splice sites 5′ and 3′ are novel). (*d*) Proportion of tags over exonic features. Tags were defined and normalized to ‘Tags/Kb’ by RSeQC (a read spliced once is counted as two tags). (*e*) Profile plot of gene body coverage depicting the proportion of coverage throughout gene bodies (scaled for all transcripts), across all platforms and differentiation days.

Using the aligned reads, we compared the general sequence quality features between the platforms. The median read length in the ONT Direct RNA platform (1615 bases) was longer than the ONT cDNA (1060) (table 1). ONT Direct RNA reads were also found to be longer in the study of Workman *et al*. [20], perhaps due to shorter transcripts bias in cDNA PCR amplification process. The average ONT error rate for the aligned reads was high, i.e. 14.3% and 11.6% in the ONT Direct RNA platform and in the ONT cDNA, respectively, in comparison to that of Illumina (0.3%), similar to the extent observed in previous reports [10,11,20,21,43]. A difference in the GC content distribution was observed (figure 2*a*; electronic supplementary material, figure S1). Illumina TruSeq reads exhibited a broader distribution (s.d. of 8.8) than the ONT reads, and broader than all GENCODE annotated transcripts (s.d. 6.2–6.4). In addition, all platforms showed a right-shift toward higher GC% values (48–50%), compared to that computed for all known transcripts (45%). To monitor whether ONT captures more novel transcripts, we examined the saturation of known and novel junctions (figure 2*b*). While all platforms reached saturation of known junctions, examination of the novel junctions showed that unlike Illumina and ONT Direct RNA, the ONT cDNA platform was farthest away from saturation. Novel junction reads can reflect the ability to capture novel transcripts, or alternatively, it can be indicative of junction mapping inaccuracies due to sequencing errors. To gain further insight, we partitioned between junctions that were detected by a single read and those detected by at least two reads, with the assumption that junctions determined by several reads are more reliable. ONT cDNA had more unannotated junctions (67%) compared to the other platforms (figure 2*c*), yet most of these complete novel (both splice sites 5′ and 3′ are novel) and partial novel (one of the splice sites 5′ or 3′ is novel) junctions were detected with a single read (52%) and are therefore less trustworthy. Analysis of read distribution over exonic features (figure 2*d*) showed that the proportion of coding DNA sequence (CDS) exons was the highest in Illumina TruSeq (0.63), and that the 5′ and 3׳ UTR exons were less represented in Illumina TruSeq in comparison to the ONT reads (0.35 versus at least 0.55 in ONT). The representation of introns and intergenic regions adjacent to annotated genes comprised a small fraction (less than 0.03) of the reads, yet the ONT platform had a higher representation (electronic supplementary material, figure S1D). In accordance with this observation, read coverage on the gene body (figure 2*e*) showed that Illumina TruSeq's coverage was significantly reduced at the 5′ and 3′ ends of the genes (*p*-values of 0.001 and 0.00025 for the 10% and 90% gene body percentiles, respectively; see Material and methods). The reduced coverage of Illumina TruSeq at the 3′ of the gene body in comparison to ONT sequencing was also demonstrated previously [24,27]. This bias can be a result of the sequencing protocol, in which transcripts are sequenced from the 3′ to the 5′ end in the ONT Direct RNA, and can be truncated due to fragmentation during the library preparation, or pore blocking during sequencing. Such 3′ bias has been shown also for ONT cDNA for the same reasons. Despite the differences in the total number of reads obtained from the different platforms (table 1), gene RPKM saturation analysis revealed a similar relative error rate per quantile of gene expression levels, upon subsampling in all three platforms (electronic supplementary material, figure S1E; from the second to the fourth quantile). The above-described quality features observed for Day4 samples were similar when analysing the Undiff datasets (electronic supplementary material, figure S1). To summarize, we have detected many differences between the platforms compared.

### 2.3. Comparison of gene expression levels

**Figure 3.**
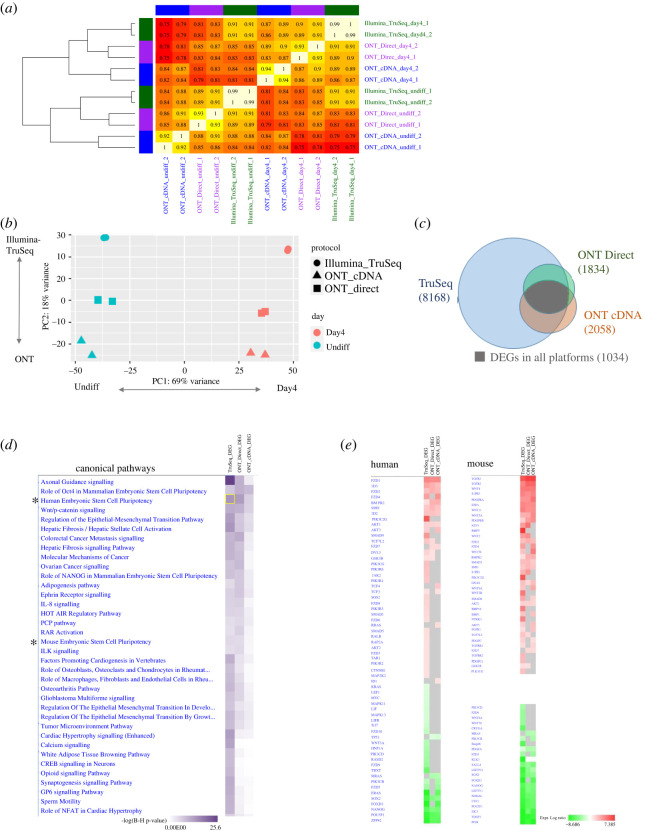
Gene-level expression analysis across the sequencing platforms. (*a*) Heatmap of Spearman correlation coefficients of raw gene counts for the Day4 and Undiff samples (in duplicates), sequenced with either Illumina TruSeq, ONT cDNA or Direct RNA. (*b*) Principal component analysis (PCA) of log-normalized values (rld) from all samples. Colours denote differentiation day and shapes denote sequencing platform. (*c*) Proportional Venn diagram of DEGs overlap, the presented numbers are the total amount of DEGs for each platform and the amount of DEGs shared by all platforms. (*d*) Ingenuity pathway analysis showing the top enriched canonical pathways, computed using DEGs from the three sequencing platforms. (*) indicates pathways elaborated in (*e*). (*e*) Ingenuity pathway analysis showing the log-fold-change expression of the top upregulated and downregulated DEGs for the human or mouse embryonic stem cell pluripotency pathways.

We next performed a gene-level analysis and observed on average, 0.6 M, 1.6 M and 36.9 M reads mapped to genes of the ONT Direct RNA, ONT cDNA and TruSeq Illumina datasets, respectively (table 1), in accordance to the raw sequencing yields. The biological replicate samples from the same platform were grouped together and exhibited high similarity (figure 3*a,b*), with a Spearman correlation coefficient in the range of 0.92–0.99. Furthermore, datasets collected at the same differentiation state from different platforms also exhibited a high correlation coefficient value, which ranged between 0.82 and 0.91 (figure 3*a*). By contrast, between different differentiation state samples (Undiff and Day4), and different platforms, a minimum correlation coefficient of 0.75 was reached. Similarly, principal component analysis (PCA) found the differentiation state to be the main source of variation, with PC1 explaining 69% of the variation, whereas PC2, which depicts the difference between the sequencing technologies, explaining 18% of the variation (figure 3*b*).

### 2.4. Detection of differentially expressed genes across differentiation states

Statistically significant DEGs between Day4 and Undiff were separately detected for each dataset (see Material and methods). In total, 1834, 2058 and 8168 DEGs were detected using ONT Direct RNA, ONT cDNA and Illumina TruSeq, respectively, indicating that the cells underwent numerous significant changes in the transition between the Undiff and Day4 states (figure 3*c*). As expected, the number of DEGs detected in the ONT datasets was lower than Illumina TruSeq, due to fewer reads and fewer aligned bases (table 1) and consequently, lower detected gene expression levels (see difference in *x*-axis scale in electronic supplementary material, figure S2). We detected 1112 DEGs overlapping in both ONT cDNA and ONT Direct RNA datasets, and most of them (1034) were also detected in Illumina TruSeq. Only two DEGs showed opposite directions among the three platforms (electronic supplementary material, table S2, Clmp and Plvap).

The three DEG sets, comparing Day4 versus Undiff, also shared similar enriched canonical pathways (Ingenuity pathway analysis; figure 3*d*), among them, the following expected canonical pathways: ‘Role of Oct4 in Mammalian Embryonic Stem Cell Pluripotency’ and ‘Embryonic Stem Cell Pluripotency’ in both human and mouse. For example, within the latter pathway, the genes FZD1, BMPR2 were upregulated and NANOG, POU5F1 were downregulated (figure 3*e*).

### 2.5. Isoform-based reconstruction and quantification of transcripts

To address our goal to detect global changes in DS during ESC differentiation, and to leverage our long read datasets, we assembled and quantified transcripts using two reference-guided methods, namely StringTie2 and TALON. Initially, TALON was used to assemble transcript models from the aligned long reads, namely Day4 and Undiff, from both ONT cDNA and Direct RNA (analysis was performed with pooled replicates). TALON identified and quantified 97 903 distinct transcript models, of them 28 575 were novel or partially novel (electronic supplementary material, figure S3A; we filtered transcripts that had less than five reads in any of the pooled samples; see Material and methods). The proportions of known transcripts detected by ONT Direct (62%) was higher than ONT cDNA (44%). Correlation between transcript abundancies (Spearman correlation coefficient) of 0.55 was observed among the ONT cDNA pooled datasets (Day4 and Undiff) and 0.82 among the ONT Direct RNA pools (electronic supplementary material, figure S3B). There was an unexpectedly negligible similarity between the platforms (ONT cDNA and Direct RNA). We also used StringTie2 [23] to quantify TALON transcripts with the 12 genome-aligned datasets (RNA-Seq mappings), and evaluated the similarity between biological replicated pairs including Illumina TruSeq datasets. Spearman correlation coefficients among the ONT replicates were in the range 0.68–0.69 and 0.84 for the Illumina replicates (electronic supplementary material, figure S3C). Between the ONT and Illumina platforms Spearman correlations were in the range of 0.1 to 0.17, for the same differentiation day, and from 0.32 to 0.41 between ONT cDNA and Direct RNA.

As a second assembly approach, StringTie2 was used on ONT and Illumina genome-aligned reads. A total of 147 769 transcripts (of them 16 852 novel) and 53 592 genes were assembled and quantified (see Material and methods; table 1). As in the TALON transcript analysis, the correlation of transcript expression among the biological replicates within the same platform was the highest for Illumina TruSeq samples (0.86), compared with ONT Direct RNA and ONT cDNA (0.63–0.66, respectively) (electronic supplementary material, figure S4A). Furthermore, the correlation coefficient between platforms was in the same range, and as low as 0.3, within the same differentiation day, implying dramatic differences in quantification across the platforms. A recent study has demonstrated that standard RNA-seq is able to robustly recapitulate only about 50% of isoforms detected by long-read Iso-Seq sequencing [44]. The lack of quantified transcript similarities between the platforms in our study and, more alarmingly, the moderate similarity between the biological replicates tested in each platform, implies that the quantified levels may not reflect the real signal, and we therefore are not describing here an isoform-based analysis, aimed to detect DS.

### 2.6. Detecting differential exon usages

**Figure 4.**
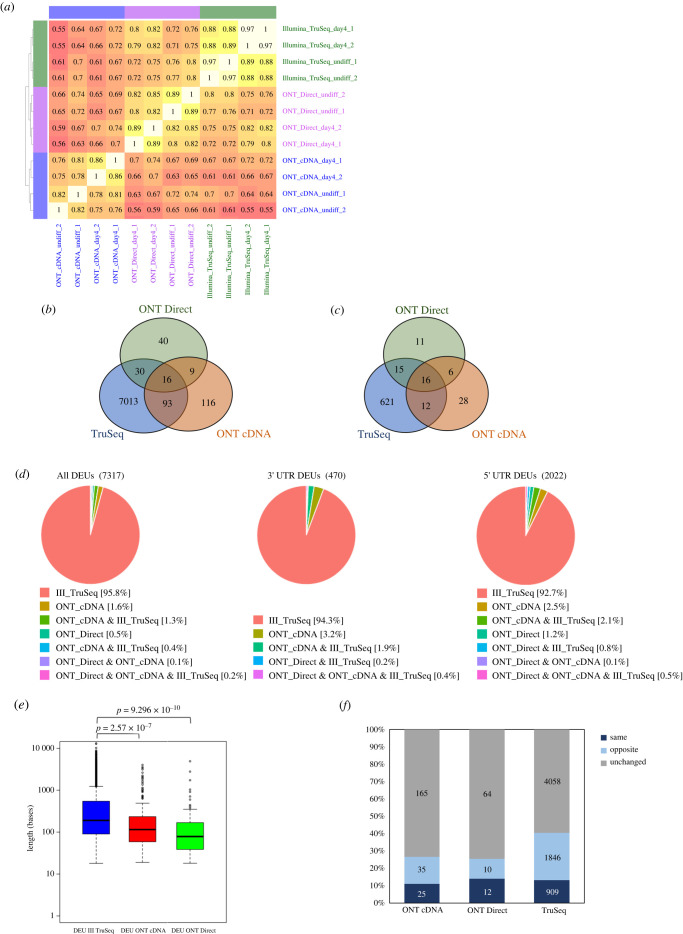
Exon-level expression analysis across the sequencing platforms. (*a*) Heatmap of Spearman correlation coefficients of raw exon counts for the Day4 and Undiff samples, sequenced with either Illumina TruSeq, or with ONT cDNA or Direct RNA. (*b*) Venn diagram depicting the overlap of DEUs detected in the different sequencing platforms. (*c*) Venn diagram depicting the overlap of DEUs, accounting only for exons which passed the expression filtering cutoffs in all platforms (see Material and methods). (*d*) Proportions of DEUs detected by the various platforms (left, a pie presentation of the Venn diagram presented in (*b*)), and specifically for DEUs in genes 3′ and 5' UTRs (right). (*e*) Box plot distribution of DEU lengths (in bases) per platform. Kolmogorov–Smirnov test *p*-values are shown. (*f*) Proportions calculated by a comparison of DEUs to DEGs, categorized by the gene: same significant trend (‘same’, both upregulated or downregulated); opposite significant trend (opposite); or the gene did not change significantly (unchanged). Proportions are shown for each sequencing platform separately, depicting the number of DEUs.

Towards our goal to detect DS, we applied an exon count-based strategy, in which exon expression levels were quantified for all the datasets and compared between the differentiation states (see Material and methods). The average total number of exons detected was 155 K for ONT and 261 K for Illumina (table 1). This was in agreement with the high number of Illumina aligned bases (on average around 4.5-fold higher). The highest similarity of exon expression values was observed between the same platform biological replicates (Spearman correlation coefficient of at least 0.82; figure 4*a*). The Spearman correlation coefficient was at least 0.76 among the samples derived from the same sequencing platform, even if they originated from different differentiation states. Yet, the correlation coefficient between platforms was lower (range 0.55–0.82), and, interestingly, ONT Direct RNA was more similar to the Illumina TruSeq dataset.

The moderate similarity in exon expression between the platforms indicates a high variance of exon detected expression between the platforms. Comparison of DEUs (i.e. changes in the relative usage of exons between the datasets) was performed using DEXSeq [45]. This is a statistical generalized linear model with the following concept: for each exon (or part of an exon) and each sample, the tool counts the number of reads that map to this exon as well as how many reads map to any of the other exons of the same gene. The ratio of these two counts, and how it changes across conditions (in this case sequencing platforms or differentiation states), infers changes in the relative exon usage (see Material and methods). Recent studies applied DEXSeq to detect DEUs with long reads [27,30]. To identify DEUs that result from a technical issue, i.e. a consequence of the sequencing platform, we ran DEXSeq on datasets collected from the same differentiation state, yet sequenced by different platforms. Running this analysis is problematic due to the significant difference in read counts between the different platforms, therefore yielding thousands of DEUs. For example, comparing the ONT Direct RNA Day4 samples to Day4 samples acquired using the other platforms, resulted in 30 423 significant DEUs. Some of these exons were visually inspected using a genome browser. One convincing example was the protein tyrosine phosphatase 4A1 gene (electronic supplementary material, figure S5), to which the platforms diverged in the coverage at both ends of the gene as well as in internal exons.

Due to the moderate similarity in exon expression between the platforms, differentiation state DEUs were sought for each platform separately. The number of exons used (passed the threshold, described in the Material and methods section) was 167 274, 46 638 and 46 418 for the Illumina TruSeq, ONT cDNA and ONT Direct RNA platforms, respectively. Overall, 7317 significant DEUs were detected in 3599 genes (figure 4*b*; electronic supplementary material, table S3), with 148 DEUs being detected in more than one platform and only 16 DEUs being detected in all three sequencing platforms. Most of the DEUs were detected only by the Illumina TruSeq dataset (7152, 97.8%). Furthermore, half of the DEUs detected by ONT were not detected by TruSeq (165 out of 329). Analysis of DEUs that passed the filtering criteria (50 counts in a least one sample) in all platforms, reveals a smaller proportion of DEUs detected only by TruSeq (figure 4*c*; 621, 88%). The reasons for the variation between the platforms are differences in the specific exon coverage as well as the expression of other exons in the gene.

Of all DEUs detected, 2889 exons contained coding sequence (CDS) regions (electronic supplementary material, table S3); 1933 were in ‘intron’ exons (exhibiting an alternative donor/acceptor site within an intron, intron retention or exon skipping, and named herein as an intron), 2022 were in 5′ UTRs and 470 were in 3′ UTRs. Interestingly, an analysis of the proportions of DEUs by exon category and platform indicated a decrease in detecting DEUs in 3′ and 5' UTRs in TruSeq relative to their percentage in all exons (figure 4*d*, decrease for DEUs detected only by TruSeq from 95.8% to 92.7%). This was in agreement with the read coverage proportions shown in figure 2*d* and the decrease in coverage of TruSeq reads towards the 3′ end of genes shown in figure 2*e*.

Exploring the DEU length distributions per sequencing platform, revealed a significant difference between the TruSeq and the ONT platforms (figure 4*e*). For instance, the expressed exons' median lengths were 190, 115 and 78 bases for TruSeq, ONT cDNA and Direct, respectively. This difference may be attributed to both the differences in read length and the assumptions underlying the mapping algorithms (i.e. STAR and minimap2).

To evaluate the relation between DEUs and DEGs, and their direction of regulation, we calculated the proportions of the DEUs to DEGs, categorized by the differential gene expression analysis information: same statistically significant trend (‘same’, both upregulated or downregulated); opposite trend, (opposite); or significantly unchanged (unchanged) (figure 4*f*). We detected that among the three platforms, at most, only 14% of DEUs showed the same statistically significant trend as in the gene-level analysis. Thus, exon-based analysis reveals many transcriptomic changes not apparent at the gene-level analysis.

Some of the genes with DEUs were already reported to exhibit DS events during mouse embryonic development. For example, Dnmt3b (DEUs found by ONT Direct RNA and Illumina TruSeq), Clk1 (DEUs identified by Illumina TruSeq and ONT cDNA) and Ctage5 (identified only by TruSeq) were found to exhibit DS in the transitional stage (from E8.0 to E9.0) [46]. Some of the genes detected only by Illumina TruSeq to have DEUs were previously reported, e.g. transcriptional initiation of a short Stra6 isoform was found in mouse ESCs in response to RA [47] and novel alternative splicing variants of Klf4 were first identified in mouse ESCs [48]. Smarcb1 was found to undergo DS in ESCs when compared with Embryoid bodies (Ebs) [49] and in our analysis in ONT Direct RNA and ONT cDNA (and was PCR validated, see next section).

### 2.7. Selection and validation of DEUs by qRT-PCR and RT-PCR

**Figure 5.**
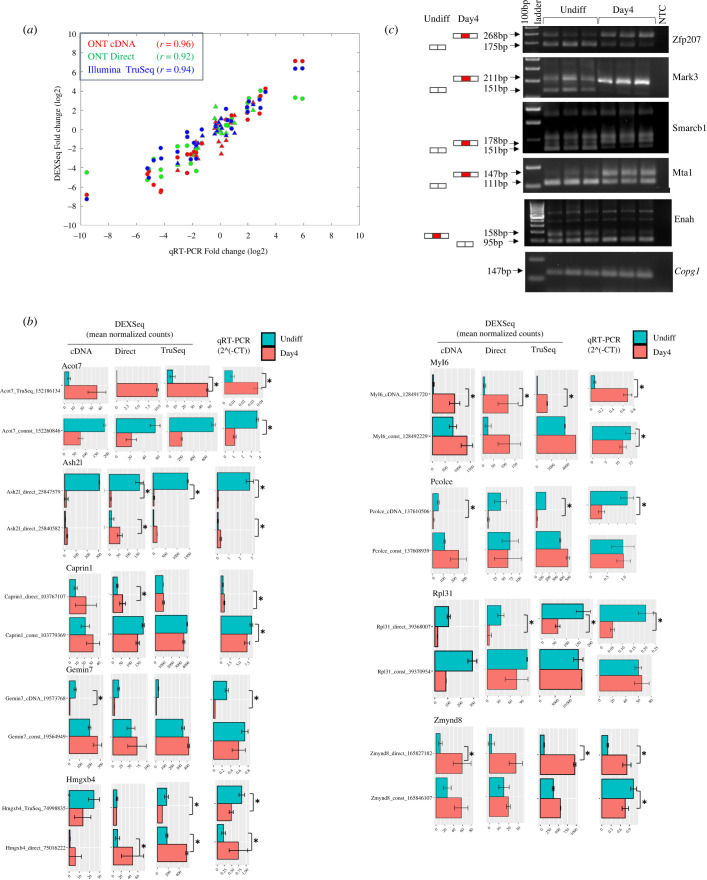
Validation of selected exons. (*a*) Scatter plot of the fold change expression ratio (Day4 versus Undiff samples) of 42 selected exons, as quantified by either qRT-PCR or DEXSeq analysis of each platform. Values represent log2 fold-change expression between the mean Day4 values and the mean Undiff values (for qRT-PCR, (2^(-ΔCт); for DEXSeq, normalized exon counts). Constitutive exons are depicted in triangles, whereas DEUs are in circles. Spearman correlation coefficients between qRT-PCR and DEXSeq log2 fold-change values are denoted. (*b*) Validation by qRT-PCR for nine genes with DEU events. Computed DEXSeq mean normalized counts (with error bars) are shown for each validated exon in each platform, along with its mean qRT-PCR 2^(-ΔCт) values (five replicates for each day, depicted by error bars). More than one exon is shown per gene, both a DEU and a constitutive exon, or two DEUs. Asterisk depicts significant changes in qRT-PCR (adjusted *p*-value = less than 0.05) between the differentiation days, or a significant DEU by DEXSeq analysis. (*c*) Validation by RT-PCR for five genes with DEU events, shown in triplicates per differentiation day. The expected products with their sizes are shown in the left panel, the alternative intronic exons are depicted in red and constitutive exons in white, arranged according to the predicted differentiation state expression preference. NTC is a negative control, Copg1 is the loading control gene.

**Figure 6.**
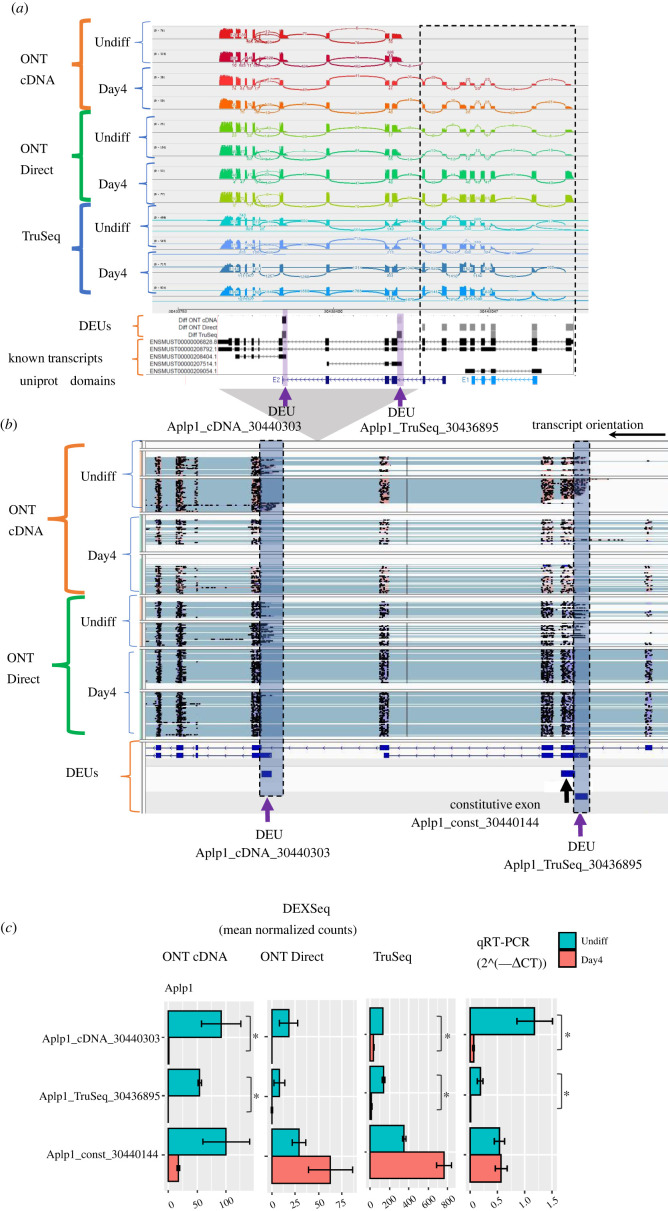
DEU analysis for the amyloid precursor-like protein 1 (Aplp1) gene. (*a*) Sashimi plot visualization of read coverage and splice junctions along the entire Aplp1 gene. Bottom tracks show DEU and known transcripts (GENCODE). In the DEU track, the greyscale is indicative of fold change (Day4 versus Undiff). The area in the rectangle marks the gene 5′-end, containing many DEUs. Purple arrows denote the DEUs selected for validation. (*b*) Visualization of ONT reads spanning the 3′-end of the Aplp1 gene. Purple arrows denote the exons selected for validation. (*c*) Validation of Aplp1 DEUs by qRT-PCR. DEXSeq exon counts as explained in figure 5.

**Figure 7.**
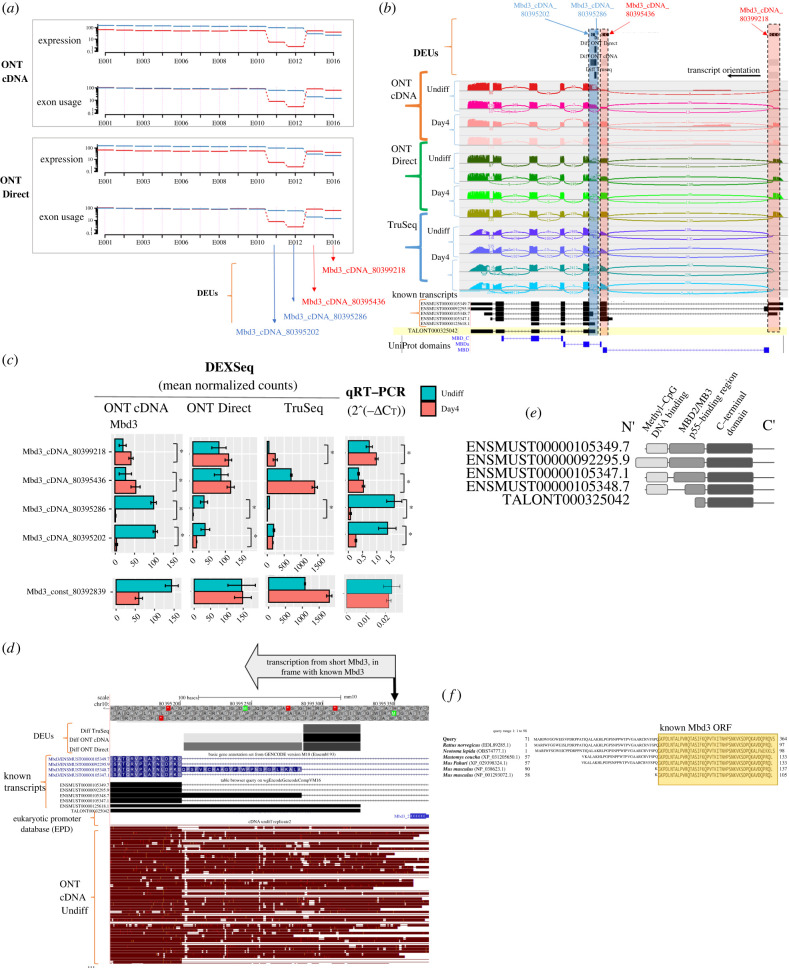
DEU analysis for the methyl-CpG binding domain protein 3 (Mbd3) gene. (*a*) Visualization of exon usage and estimated expression of Mbd3 gene exons from DEXSeq analysis results, for Day4 (red) and Undiff (blue) samples, sequenced by ONT cDNA and Direct RNA platforms. The arrows denote the DEUs selected for validation. (*b*) Sashimi plot visualization of read coverage and splice junctions along the Mbd3 gene. Top track: Predicted DEUs. Bottom track: known Mbd3 transcript models, and a novel transcript predicted by the TALON pipeline. (*c*) Validation of Mbd3 DEUs by qRT-PCR, as explained in figure 5. (*d*) Top tracks show the alternative promoter and DEUs. Subsets of ONT cDNA Undiff reads starting approximately at the alternative transcription start site (TSS), which may encode for an ORF starting at the depicted methionine (see top arrow pointing downwards). In the DEU track, the greyscale is indicative of fold change (Day4 versus Undiff). A plot of all ONT cDNA reads is shown in electronic supplementary material, figure S8. (*e*) Schematic domain architecture of the protein products of either known or predicted TALON transcripts. Pfam domains from left to right: Methyl-CpG binding domain (PF01429), MBD2/MB3_p55 binding domain (PF16564) and C-terminal domain of methyl-CpG binding protein 2 and 3 (PF14048). (*f*) Multiple sequence alignment of the protein sequence encoded by the alternative Mbd3 TSS, starting at the above depicted methionine (*d*), with orthologue protein sequences.

Initially, we explored the correlation between the calculated DEXSeq exon expression-fold changes (Day4 versus Undiff) in the three platforms, and their observed qRT-PCR-fold change values. Towards this aim, qRT-PCR was performed on 26 DEUs from 19 different genes (Acot7, Aplp1, Ash2 l, Caprin1, Egfl7, Gemin7, Hmgxb4, Mbd3, Mta1, Myl6, Nfu1, Pcolce, Rpl31, Rps24,Tmsb10, Serf2, Usp7, Wbp1, Zmynd8, see electronic supplementary material, table S3), and 15 constitutive exons (i.e. exons which did not exhibit differential usage) from the same genes (electronic supplementary material, tables S4 and S5A). The selected DEUs were calculated to be significant by either one (seven exons) or more of the platforms: 14 by ONT cDNA, 13 by ONT Direct RNA and 18 by Illumina TruSeq. Some of the DEUs (13 exons) were in the coding sequence and protein motifs (four exons), thus presumably affecting the protein function (electronic supplementary material, table S3), and some were in ‘introns’ (14 exons). The observed qRT-PCR-fold change values were highly correlated (Spearman correlation coefficient of at least 0.92) to their calculated DEXSeq-fold changes (figure 5*a*). To validate the DEUs, we selected more than one exon per gene, either a DEU or a constitutive exon or at least two DEUs (excluding the genes Serf2 and Tmsb10; see Material and methods). The experimental qRT-PCR mean expression values of DEUs from 11 genes along with their calculated DEXSeq values demonstrated that these DEUs significantly changed between the differentiation days, while the additional exon from the same gene showed an opposite trend or an insignificant change (figures 5*b*, 6*c* and 7*c*). For instance, the gene Hmgxb4 had a significantly high expression of the DEU named Hmgxb4_TruSeq_74998835 (detected as a significant DEUs in TruSeq) in the qRT-PCR Undiff samples, whereas the additional DEU named Hmgxb4_direct_75016222 (detected as a significant DEU in TruSeq and Direct) had a significantly high expression in qRT-PCR Day4 samples (figure 5*b*). In five additional genes, both the DEU and the constitutive exon were upregulated in the same differentiation state but not to the same extent, as evident by their log2 fold-change of Day4 versus Undiff by both DEXSeq and qRT-PCR (electronic supplementary material, figure S6 and table S4). We present also the gene Rps24, in which the exon expression values were in the trend expected, yet in order to better validate the DEU, a different constitutive exon should have been selected (electronic supplementary material, figure S6 and table S4, not considered as validated).

As a second validation approach, we performed RT-PCR to confirm DEU events of alternative splicing in ‘intronic’ coding exons within the genes: Enah detected by TruSeq and Zfp207, Mark3, Smarcb1 and Mta1, detected by ONT Direct (one DEU was detected also by TruSeq and two others also by ONT cDNA). RT-PCR was performed using primer pairs designed to target the immediately flanking constitutive exons, and detected the two expected amplified product sizes, in accordance to the differentiation day (figure 5*c*; electronic supplementary material, table S5B). The presence of additional bands can be explained by additional isoforms or non-specific amplification. To summarize, we validated DEUs from 20 genes by qRT-PCR and RT-PCR, predicted by DEXSeq analysis in one or more of the sequencing platforms.

### 2.8. Exploring DEUs in the Aplp1 gene

An example of a gene that exhibited DEUs was the amyloid-like protein 1 (Aplp1) gene, belongs to a family of proteins involved in neuronal development and in dementia [50]. This gene was also identified as a DEG only by ONT cDNA. Overall, we identified 12 DEUs within this gene, many of them are in the 5′ end of the gene and were identified mostly by ONT Direct in a region of the gene that contains the ‘E1’ domain (figure 6*a*; electronic supplementary material, table S3). The Aplp1 gene harbours transcripts (for example: ENSMUST00000207514.1 and ENSMUST00000208404.1) that might not encode a protein, and have alternative transcription start sites (TSS). Some of the ONT reads start at these TSS, in addition, we identified two DEUs (Aplp1_cDNA_30436895 and Aplp1_cDNA_30440303; figure 6) that overlap these alternative TSS and were significantly upregulated in the Undiff state, as detected by the TruSeq and ONT cDNA platforms. These DEUs were validated by qRT-PCR and their expression was demonstrated to be significantly different between Undiff and Day4, as expected (figure 6*c*; electronic supplementary material, table S4).

In an attempt to decipher differentially expressed transcript models containing and starting at the two validated DEUs, we explored the StringTie2 assembled and quantified transcripts (electronic supplementary material, figure S4B). The analysis revealed two short transcripts that were highly expressed in the Undiff state within the ONT cDNA datasets. Similarly, TALON assembled two short transcripts with a TSS similar to ENSMUST00000208404.1, starting with the DEU Aplp1_cDNA_30440303, which were more abundant in Undiff. Yet, no evidence was found for a transcript starting at the DEU Aplp1_cDNA_30436895 (data not shown).

### 2.9. Exploring DEUs in the Mbd3 gene

Gene-level analysis did not identify the Mbd3 (methyl-CpG binding domain protein 3) gene as a DEG between Undiff and Day4 samples, yet exon-based analysis identified DEUs within this gene. Mbd3 is an essential pluripotency gene, and is a key component of the NuRD chromatin remodelling complex [51,52]. Four Mbd3 gene DEUs were detected by either one or more platforms (figure 7; electronic supplementary material, figure S7). In GENCODE, these are three exons, however, in the analysis, one exon was split into two since there were transcripts in which this exon was only partially overlapping. Two of the exons were upregulated in Undiff (Mbd3_cDNA_80395202 and Mbd3_cDNA_80395286 are in fact part of the same exon), and two were upregulated in Day4 samples (Mbd3_cDNA_80395436 and Mbd3_cDNA_80399218; figure 7*a*,*b*). These exons, along with a constitutive exon were selected for validation by qRT-PCR (figure 7*c*), and their expression was demonstrated to be significantly different between Undiff and Day4, as expected.

ONT reads revealed an annotated transcript start of a short Mbd3 isoform, presumably upstream to the DEUs upregulated in Undiff (Mbd3_cDNA_80395202 and Mbd3_cDNA_80395286) (figure 7*d*; electronic supplementary material, figure S8). Spliced isoforms for Mbd3 are reported in the sequence databases, and result in different protein products (figure 7*e*). Specifically, the *N*-terminal methyl-CpG binding and MBD2/MB3_p55 binding domains are shorter in the known transcripts (i.e. ENSMUST00000105347.1; figure 7*e*), or entirely missing in ENSMUST00000125618.1 (annotated as non-coding, therefore not shown in figure 7*e*). Yet, the above annotated isoforms initiate downstream to our ONT reads (figure 7*d*). The ONT reads initiate proximal to a reported alternative promoter (located at chr10:80 395 362–80 395 421; EPDnew UCSC track in figure 7*d*), that suggests the presence of an additional mechanism for transcriptional regulation. A second indication for the above TSS is that its 5׳ exon encodes an ORF in frame with the Mbd3 gene, extending by 49 amino acids an internal exon encoding the MBD2/MB3_p55 binding domain (sequence detailed in electronic supplementary material, file S1). Part of this ORF (28 amino acids) overlaps an exon in ENSMUST00000105348.7. An alignment generated by TBLASTX with the 5′ end of our predicted shorter transcript, showed that the 49 amino acids were conserved in other species, such as *Rattus norvegicus* (GenBank: EDL89285.1) (figure 7*f*).

Even though this isoform does not exist in the public sequence repositories, experimental support for the functionality of this alternative transcript was reported by Ee *et al*. [53]. They demonstrated mouse ESCs expression of an Mbd3 isoform (Mbd3C) bearing a unique 50-amino-acid N-terminal region that is necessary for interaction with the histone H3 binding protein WDR5. This interaction creates a unique NuRD complex variant that specifically functions in ESCs.

We further explored the presence of the novel short Mbd3 and its differential upregulation in the Undiff state, within our transcript assembly datasets. No Mbd3 novel transcript was detected in the StringTie2 assembly. Furthermore, transcript expression plots for the Mbd3 gene did not reveal a coherent transcript expression pattern between the replicates or between the platforms (using StringTie2 FPKM quantification; electronic supplementary material, figure S4C). Therefore, StringTie2 did not support transcript models explaining the validated DEUs. By contrast, in TALON-assembled transcripts predicting for the Mbd3, we identified five known isoforms and seven novel transcripts with at least five supporting reads (electronic supplementary material, figure S9). One of the detected novel transcripts (TALONT000325042), initiated downstream to the alternative TSS described above, was 10-fold more abundant in Undiff in both ONT platforms (figure 7*b*,*d*,*e*; electronic supplementary material, table S6).

## 3. Discussion

This work aimed to detect transcriptome changes in the gene, transcript and exon levels using three sequencing platforms, namely Illumina short reads, ONT cDNA and ONT Direct long reads, towards the discovery of DS events in mouse ESCs undergoing differentiation.

At the gene level, the differentiation state was the dominant cause for variations in gene expression values. While there was a high overlap in the DEGs detected by the sequencing platforms and all DEG lists were enriched for ESC pluripotency pathways, the Illumina technology had an advantage in detecting more DEGs due to the higher number of reads and thus higher transcriptome coverage.

In contrast to the gene level, in the isoform-based analysis the agreement in expression levels between platforms decreased, comparing transcripts assembled and quantified (using TALON and StringTie2) across the platforms resulted in a weak or non-detectable correlation. Given the high number of expected transcripts assembled (greater than threefold than the number of genes), it is likely that the low ONT read yield imposes a limitation to accurately quantify transcripts. Thus, despite the premise of long reads in constructing transcripts, their low throughput and low sequence accuracy demonstrate that generating accurate transcriptomes from imperfect RNA reads is still a challenge [22,31]. Moreover, it is hard to distinguish whether reads with premature starts and ends indicate native internal transcription start or end sites, or technical issues such as fragmented reads or blocked pores. This phenomenon inflates the number of reconstructed transcripts. Nevertheless, they can also be genuine alternative TSSs, as we demonstrated for the Mbd3 gene that are upregulated in the Undiff state, and encode a shorter Mbd3 protein that lacks the *N*-terminal methyl-CpG DNA binding domain.

We have shown that each of the technologies presents various sequence biases, which is a consequence of differences in reads length, number of reads, error rates, biased coverage along the gene body and the ability to accurately detect exon junctions. These biases can explain the low reproducibility of reconstructed transcripts quantification. A recent hybrid approach uses both short and long reads to improve the final reconstructed transcripts set [27]. However, sequencing biases that we observed (i.e. decreased coverage of the short reads at the transcript UTRs) still need to be addressed in the hybrid approach. In addition, the short reads cannot aid in identifying true internal transcription start or end sites. Ongoing advances in computational algorithms increase the accuracy of sequence and isoforms detection, such as isONcorrect method described by Sahlin *et al.* [21], and is applicable to cDNA long-read sequencing. Furthermore, technology advancements have improved ONT cDNA sequencing accuracy by implementing rolling circle amplification to concatemeric consensus (R2C2) method [43,54,55] and by ONT latest chemistry (kit 12 chemistry) and R10.4 pores, which enables 99.3% raw read accuracy. Yet, all the above-described advancements are not applicable to Direct ONT sequencing.

The exon-based strategy enabled the detection of numerous statistically significant DEUs, by both short and long sequencing platforms. Interestingly, most of DEUs were detected uniquely by one of the platforms: the long reads exhibited an advantage in detecting DEUs at the UTRs, whereas short reads had an advantage in detecting an order of magnitude more DEUs than with the ONT platforms. The fact that most of the DEUs were not found in genes detected as DEGs, suggests that numerous DS events occur during ESC differentiation, and could not be detected at the gene-level analysis. Only a fraction of DEUs were detected by all platforms (16 out of 7317), still, DEUs from 20 genes (detected by one or more platforms) were validated by qRT-PCR and RT-PCR, including DEUs that are protein coding and alternatively spliced introns in key genes of the ESCs differentiation process (Mbd3 and Aplp1), or in chromatin modification (Ash2 l, Mbd3, Mta1, Smarcb1, Usp7) as well as others. Taken together, we suggest the exon-based approach as a promising strategy for deciphering DS events, furthermore, we highlight that the sequencing platforms reveal complementary information. In summary, this work provides an extensive repository of short and long reads, along with gene and exon-based analyses, profiling the transcriptomic changes upon RA-induced mouse ESC differentiation, which can be used as a resource for discovering functional diversity.

## 4. Conclusion

In this study, we explored three sequencing platforms, namely Illumina short reads, ONT cDNA and ONT Direct long reads, and found that at the gene-level expression, Illumina short reads identified more changes due to its higher sequencing yield, yet the three platforms discovered similar transcriptomic profiles. In an attempt to discover DS during mouse ESC differentiation, quantification of reconstructed transcripts was found to be irreproducible. Thus, even with the use of long reads, precise transcript structure reconstruction and transcript quantification remain challenging, due to the low read yield and accuracy. We hereby demonstrated that exon-based strategy can bridge this challenge and detect statistically significant DEU, by both short and long sequencing platforms, and that the three sequencing platforms complement one another. In addition, we provide an important analysed resource of transcriptome changes occurring during stem cell differentiation.

## 5. Material and methods

### 5.1. Cells and RNA extraction

R1 mouse embryonic stem (mES) cells were maintained in mES growth medium (DMEM, fetal bovine serum, L-glutamine, non-essential amino acids, penicillin/streptomycin, ß-mercaptoethanol and leukaemia inhibitory factor), and named herein as sample Undiff. Embryoid bodies were generated from R1 single-cell suspensions (35 000 cells ml^−1^) in mES growth medium without Leukaemia inhibitory factor in low adherence dishes and grown for 4 days. Thereafter, they were treated for 4 days with 2 µM RA (Sigma R2625), and termed sample Day4. The experiment was conducted in two biological replicates from distinct samples that were grown and treated separately. RNA was extracted using the RNeasy Mini Kit (Qiagen), and its quality was assessed using TapeStation (Agilent). Poly(A) RNA was isolated from the total RNA using the Dynabeads mRNA DIRECT kit (ThermoFisher Scientific) according to the manufacture's protocol.

### 5.2. Illumina library preparation and sequencing

A total of 1 µg RNA was processed using the Illumina TruSeq RNA Sample Preparation Kit v. 2 protocol. Libraries were evaluated by Qubit and TapeStation. Sequencing libraries were constructed with barcodes to allow multiplexing. Between 39 and 59 million paired-end reads were sequenced (2 × 101 bases) per sample (table 1), on Illumina HiSeq Rapid 2500 instrument using protocols RTA (1.17.21.3) and HCS (2.0.12.0).

### 5.3. ONT library preparation and sequencing

For cDNA-PCR library preparation, a total of 50 ng poly(A) RNA was used as input. Libraries were prepared according to manufacturer's protocols using the cDNA-PCR Sequencing Kit (SQK-PCS108, ONT, Oxford, UK; one dimension—meaning that the template and the complement strands are sequenced as individual strands). Input of 500 ng poly(A) RNA was used for the Direct RNA library preparation kit (SQK-RNA002, ONT, Oxford, UK). Both types of libraries were sequenced using the ONT MinION 106D R9 version flow cells. MinKNOW software (v. 3.1.8, ONT) was used to run each flow cell for 48 h.

### 5.4. Quantitative real-time reverse transcription polymerase chain reaction

Complementary DNA was synthesized from 500 ng total RNA using the PrimeScript RT Reagent Kit (TAKARA) according to the manufacturer's instructions, with both oligo-dT primers and random hexamers. qRT-PCR primers were designed for DEUs and constitutive exons (for the genes Serf2 and Tmsb10, only one DEU was designed and no constitutive exon was selected) using either primer3 (https://bioinfo.ut.ee/primer3-0.4.0/) or Blast-Primer (https://www.ncbi.nlm.nih.gov/tools/primer-blast/) and are listed in electronic supplementary material, table S5A. qRT-PCR analyses were performed with SYBR Green (Applied Biosystems, Foster City, CA, USA) on five replicates per exon and differentiation day. Signals (cT) were normalized to genes and exons that were found to have high and similar expression levels in all platforms and differentiation days (Pum2, Sec24d, Copg1). To compare between Day4 and Undiff, we performed a *t*-test on 2^(-ΔCт) of Day4 versus Undiff, and *p*-values were corrected with a Benjamini–Hochberg adjustment. Fold-changes were calculated between mean Day4 values 2^(-ΔCт) and mean Undiff values.

### 5.5. Reverse transcription polymerase chain reaction

RT-PCR primers were designed from constitutive exons that flank DEUs classified as introns and yield an amplified product less than 500 bp (primers are listed in electronic supplementary material, table S5B). The Copg1 gene was used as a loading control. cDNA was prepared as described above, and PCR was done using the primers and KAPA Hifi HotStart ReadyMix (Roche; Cape Town, South Africa). The cycling acquisition programme used was: initial denaturation at 98°C for 2 min, followed by 35 cycles of denaturation temperature at 98°C for 20 s, annealing at 64°C for 30 s and elongation at 72°C for 30 s; and a final elongation step at 72°C for 1 min. RT-PCR products, assayed in triplicates per differentiation state, were resolved using 2% SeaKem LE (Lonza, Rockland, ME, USA) agarose gel.

### 5.6. Genome browsers

The UCSC genome browser [56] (https://genome.ucsc.edu/s/bareket/mm10%2DES%2Dtranscriptome%2Danalysis) and IGV v. 2.9.4 [57] were used to visualize the reads and the assembled transcripts on selected genomic regions. Searching for protein sequence similarities with novel Mbd3 transcript start was performed by running TBLASTX against the nucleotide database (nt) at NCBI (https://blast.ncbi.nlm.nih.gov/).

### 5.7. Bioinformatics analysis of Illumina sequences

Raw reads were analysed using the UTAP transcriptome analysis pipeline [58]. Initially, reads were trimmed using cutadapt v. 1.15 [59] to remove TruSeq adaptors, with the parameters: -times 2 -q 20 -m 25. Reads were mapped to the *Mus musculus* genome (mm10, GENCODE annotation) using STAR (v. 2.4.2a) [60], with the following parameters: -alignEndsType EndToEnd, -outFilterMismatchNoverLmax 0.05 and -twopassMode Basic.

### 5.8. Bioinformatics analysis of ONT sequences

Direct RNA reads were acquired using the MinION software from Oxford Nanopore Technologies (ONT), and base-called using either ONT albacore (MinKNOW v. 2.3.1 and 2.3.3) or Guppy software v. 2.1.3 (electronic supplementary material, table S1). Raw reads were converted from fast5 to fastq format and processed to base calls using Poreplex v. 0.3.1 and 0.4.1 (https://github.com/hyeshik/poreplex), trimmed to remove any 3′ adapter sequences, and filtered to remove chimeras (unsplit reads fused of two or more RNAs), with the parameters -trim-adapter -basecall -filter-chimera.

ONT cDNA raw reads from the ‘skip’ folder were base-called using Guppy (–flowcell FLO-MIN106 –kit SQK-PCS108). These reads were merged with reads from the pass folders, and processed using porechop (v. 0.2.3) to remove adaptors.

The pre-processed reads were aligned to the *Mus musculus* genome (mm10, UCSC) using minimap2 (v. 2.10) adjusted for long-read spliced alignment (-x splice, -secondary = no, -MD). SAM alignment files were sorted, and converted to indexed BAM files. For DEXSeq analysis, the resulting primary aligned reads were marked with ‘NH:i:1’ tags using the UNIX awk command. The per cent of reads multiple aligned was below 2.4%.

### 5.9. Gene-level analysis

Reads on genes were counted using htseq-count [61] with mm10 annotation (downloaded from iGenomes UCSC), and considering the strandedness of the samples (Direct RNA samples were run as strand-specific; -s yes).

Spearman correlation coefficient analysis was performed on the raw counts using the cor function in the R stat package [62], and heatmaps were created using the gplots package (heatmap.2). PCA analysis was performed on log-normalized values, computed with DESeq2 (rlog function, blind = TRUE) [63] using the R prcomp package.

Differential gene expression analysis was performed separately on the count matrix for each of the platforms, using the UTAP pipeline [58]. Specifically, normalization of the counts and differential expression analysis were performed using DESeq2 (v. 1.16.1) with the parameters: betaPrior=True, cooksCutoff = FALSE, independentFiltering = FALSE. The following criteria were used to select DEG: adjusted *p*-value ≤ 0.05, |log2FoldChange| ≥ 1 and baseMean ≥ 5.

Enrichment analysis of canonical pathways of the three DEG lists along with their log-fold change values (Day4 versus Undiff) was performed using Ingenuity Pathway Analysis (Qiagen, 2021, https://digitalinsights.qiagen.com/products-overview/discovery-insights-portfolio/analysis-and-visualization/qiagen-ipa/). The DEG lists enrichment results were compared using the ‘comparison’ feature in IPA, using both human and mouse, in order to benefit from the rich pathway knowledge of both organisms.

### 5.10. Comparison of sequence quality control features

Various sequence quality control features were extracted from the aligned reads (separate BAM file for each sample and platform) using the RSeQC tool v. 2.6.4 [64] and NanoPlot v. 1.14.1 [65]. The features extracted were GC content per read (read_GC.py), read distribution over genome regions (read_distribution.py), junction novelty (junction_annotation.py), junction saturation (junction_saturation.py), genebody coverage (genome_coverage.py) and RPKM saturation (rpkm_saturation.py, ONT Direct RNA was run with –d ‘++,–’). The read coverage analysis on gene body was done by scaling all transcripts to 100 bases and calculating the proportions of reads covering each nucleotide position. Statistical significance was calculated for specific gene body percentile coverage (10%, 90%) using ANOVA. The RSeQC output files per sample were merged using R and Excel to a single plot, and in some of the plots, the replicate values were averaged before plotting. GC content of the transcriptome was calculated using bedtools nuc on all transcripts [66]. Alignment error rates were calculated using AlignQC v. 2.0.5, read bases aligned were counted using samtools v. 1.12 (stats, selecting ‘bases mapped (cigar)’) [67].

### 5.11. Exon-count-based analysis

The GENCODE annotation gtf file vM15 was processed by running the python script dexseq_prepare_annotation.py (option -r no). The exons were quantified with dexseq_count.py using options:’ -r pos -s no’, except for ONT Direct RNA that ran with the option ‘-s reverse’, and ‘-p yes’ for Illumina TruSeq. Spearman correlation coefficient analysis of the raw counts of all exons (merged using DESeq2 DESeqDataSetFromHTSeqCount) was as described above for the gene-level analysis.

DEUs between Undiff and Day4 (or the day attribute) samples was detected using R 3.5.1 and DEXSeq R package (v. 1.26.0) [45], run separately for each platform. Initial filtering was performed to keep exons that have 50 counts in a least one sample, and at least 10 reads in ‘other exons’ in all samples. Differentially used exons were found using the DEXSeq function testForDEU using the full model (fullModel = sample + exon + day:exon), and a reduced model (reducedModel = ∼ sample + exon). Analysis of exons that are differentially expressed between the sequencing platforms for a certain differentiation state (either Undiff or Day4), was performed by including the platform category in the model (instead of the day). Criteria for selecting differentially expressed exons were adjusted *p*-value ≤ 0.05 and an absolute |log2FoldChange| ≥ 1, and a minimal exon length of 18 bases. For validation we also included an exon from the gene Wbp1, ENSMUSG00000030035.14:E016, that had an adjused *p*-value of 0.09. Annotation of the DEUs (between Undiff and Day4) into 5′ UTR, 3′ UTR, CDS or intron (meaning alternatively spliced intron) categories was done by running bedtools intersect (parameters -s -u) using a BED file of the DEUs and exon category-derived GTF files. These GTF files were derived using the R package GenomicFeatures [68] and GENCODE (vM15) annotation. The outputs were intersected with the original GENCODE GTF to add the gene_id using bedtools [66] (parameters: intersect -s -wa -f 1.0). Annotation of protein domains was done by intersection with UCSC table unipDomain (release 2020_06).

### 5.12. Quantifying and characterizing isoform-level expression of genes with TALON

The TALON package [22] was applied to identify and quantify isoforms in ONT samples (cDNA and Direct RNA). The alignments, pooled from both replicates, were pre-processed with talon_label_reads to remove artefacts of internal priming with A-rich sequences (20 bp window). The TALON database was initialized from the GENCODE (vM15) annotation with talon_initialize_database module (parameters: –l 0 –5p 500 –3p 300). The TALON module was applied for transcript annotation (parameters: –cov 0.9 –identity 0.8), keeping transcript models with greater than 5 reads in at least one of the pooled replicates. Overall, each pool identified less than 48.3 K distinct transcripts that were merged to a total of 97 904 distinct transcript models (merged GTF) (electronic supplementary material, figure S3A). A Spearman correlation was calculated between the transcript abundances (quantified using talon_abundance function) for the pooled replicates. In addition, the pooled transcripts (merged GTF) were quantified for each sample (using the aligned reads), including the Illumina TruSeq genome-aligned sequences using StringTie2 (see details below for parameters). The FPKM values from the t_data.ctab file outputs were used to calculate Spearman correlation coefficients.

### 5.13. Transcript assembly and quantification with StringTie2

StringTie2 [23] v. 2.1.4 was used to run guided assembly with the GENCODE (vM15) annotation (parameter -G -B) from the aligned reads. The parameter (-L) was implemented for the ONT reads and the parameter (–rf) for the ONT Direct RNA reads. The transcripts were then merged to one gtf file (–merge) and estimates of transcript abundance were done by running StringTie2 with the addition of the parameter (-e). Overall, 147 769 distinct transcript models were identified. Spearman correlations were calculated on the transcripts FPKM counts. Expression plots were prepared with the R package ballgown v. 2.22.0 [69] function plotTranscripts using FPKM measures.

### 5.14. Protein domain analysis

Protein domains in Mbd3 isoforms were inferred by DoChaP [70] and Pfam database searches [71].

## Data Availability

Custom tracks are available on the UCSC browser using the session: https://genome.ucsc.edu/s/bareket/mm10%2DES%2Dtranscriptome%2Danalysis. The data discussed in this publication have been deposited in NCBI's Gene Expression Omnibus and are accessible through GEO Series accession number GSE156371. The data are provided in electronic supplementary material [72].
